# Glycosphingolipids in human parasites

**DOI:** 10.1002/2211-5463.13662

**Published:** 2023-06-23

**Authors:** Richard D. Cummings

**Affiliations:** ^1^ Division of Surgical Sciences, Department of Surgery, Beth Israel Deaconess Medical Center Harvard Medical School Boston MA USA

**Keywords:** antibodies, glycans, glycolipids, glycosphingolipids, oligosaccharides, parasites

## Abstract

Glycosphingolipids (GSLs) are comprised of glycans (oligosaccharides) linked to a lipid containing a sphingosine moiety. They are major membrane components in cells of most animals, and importantly, they also occur in parasitic protozoans and worms that infect people. While the endogenous functions of the GSLs in most parasites are elusive, many of these GSLs are recognized by antibodies in infected human and animal hosts, and thus, their structures, biosynthesis, and functions are of great interest. Such knowledge of GSLs could lead to new drugs and diagnostics for treating infections, as well as novel vaccine strategies. The diversity of GSLs recently identified in such infectious organisms and aspects of their immune recognition are major topics of this review. It is not intended to be exhaustive but to highlight aspects of GSL glycans in human parasites.

## Graphical abstract

The parasites that infect humans and animals include protozoans and helminths (worms). Their secretions and cellular membranes contain complex glycomolecules including glycoproteins and glycolipids. A class of glycolipids shared between humans and parasites are the glycosphingolipids (GSLs) containing a lipid (ceramide) linked to a glycan (oligosaccharide). This review describes these GSLs and their functions and recognition by antibodies in infected individuals.

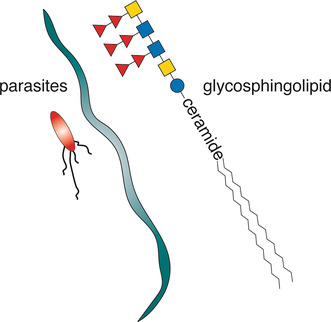

**Abbreviations**
Cer: ceramide
GPI: glycosylphosphatidylinositol
GSL: glycosphingolipid
PC: phosphorylcholine

Glycolipids are glycomolecules in which carbohydrates are linked to lipid moieties. They are found in all five kingdoms of life—animals, plants, fungi, protist (amoeba), and monera (prokaryotes). The lipid and carbohydrate moieties, however, can vary greatly depending on the organism and its cellular structures. For example, Gram‐negative bacteria express lipopolysaccharides, which are typically colossal polysaccharides linked to a core lipid A moiety that contains a wide variety of acyl chains linked to glucosamine [1]. Plants, bacteria, and algae can express a wide variety of glycolipids, including those in which sugars, such as galactose, are linked to diacylglycerol [2]. But the major focus of this review is a class of glycolipids termed glycosphingolipids (GSLs), which are defined as sugars linked to ceramide, which contains the lipid sphingosine and a fatty acid [3] (Fig. 1).

**Fig. 1:**
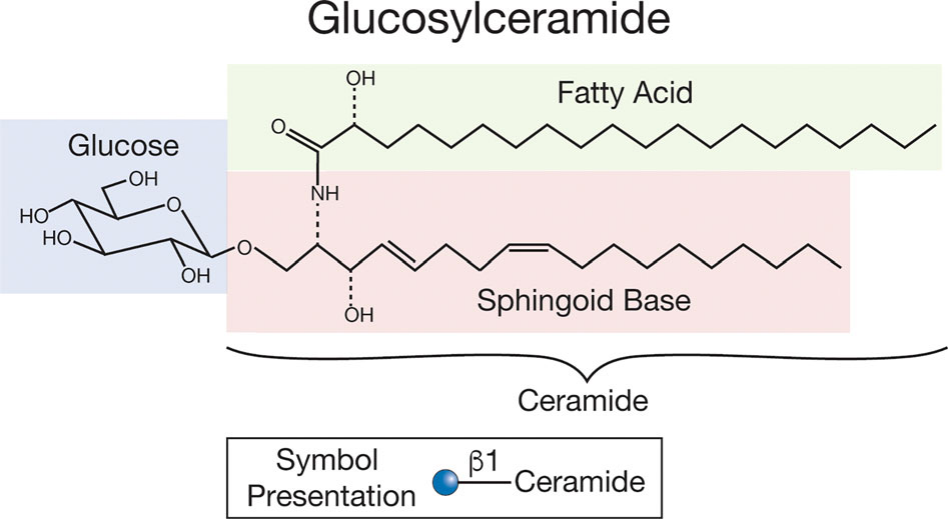
Depicted is a typical GSL that contains ceramide comprised of sphingosine and a fatty acid and linked by a glycosidic bond to a sugar. The sugar in this example is glucose as in glucosylceramide (or cerebroside), a very common GSL found in many organisms and is a precursor to more complex GSLs. The structures of the fatty acid and the sphingoid base vary depending on the source of the GSL.

Glycosphingolipids are synthesized by vertebrates and invertebrates and are also found in some species of bacteria [3]. The GSLs have many biological functions, such as modulating cellular growth, cellular interactions, and differentiation. Additionally, in animal cells they are also binding sites for many pathogens and their toxins, for example, cholera toxin and Shiga toxin [4]. But here, the focus will be on the expression of GSLs in parasites that infect humans, but mention will also be made of those infecting animals, as many parasites use animals as intermediate hosts. There are many types of parasites but the GSLs in only a few of them have been well studied in terms of structures, biosynthesis, and functions. Parasites also express other types of glycolipids such as glycoglycerolipids and glycosylphosphatidylinositol (GPI)‐anchored glycoproteins, but these types of glycolipids will not be considered here.

## The types of parasites that infect animals and humans

Parasites are eukaryotic organisms that infect us and animals and include three major types—unicellular protozoans, multicellular parasites including helminths (worms), and ectoparasites [5]. They cause tremendous suffering and death worldwide, and there are no effective vaccines against the many types of parasites that infect humans [6, 7, 8]. Examples of human parasites include protozoans such as *Plasmodium falciparum* that causes malaria, *Trypanosoma* spp. that cause Chagas disease, and *Leishmania* spp. that cause leishmaniasis. Examples of helminths include nematodes (roundworms), for example, *Ascaris lumbricoides* that causes ascariasis, *Ancylostoma duodenale* and *Necator americanus* that cause hookworm infections, and *Wuchereria bancrofti* that causes lymphatic filariasis; cestodes (tapeworms), for example, *Echinococcus multilocularis* that causes alveolar echinococcosis; trematodes, for example, *Schistosoma mansoni* that causes schistosomiasis; and tapeworms, for example, *Taenia solium* that causes taeniasis. Examples of ectoparasites include lice and bedbugs. While we commonly do not think of bacteria as parasites, there are bacteria that are obligate intracellular parasites, for example, *Rickettsia* and *Chylamydia*, that live inside eukaryotic cells. The parasitic protozoans and helminths not only express many types of GSLs and other glycolipids depending on the organism, but also can use our glycolipids in their process of infection and immune evasion.

## General structures of GSLs

The variation in the carbohydrate and lipid moieties of GSLs is tremendous and their structures are dictated by genetic pathways regulating the production of glycosyltransferases to construct the carbohydrate moieties and their additional modifications with aglycones, such as sulfate or phosphate, and lipid modification pathways that construct the ceramides. A common GSL found in many parasites and humans is glucosylceramide, also termed glucocerebroside (Fig. 1); it is the precursor to many other types of GSLs with extended glycans emanating from glucose. The GSLs are typically expressed on membranes of the secretory pathway and enriched in the plasma membranes where they are often concentrated in membrane microdomains, such as lipid rafts, and GSLs also occur in exosomes generated from cell membranes. Indeed, gangliosides in animal cells, which are sialic acid‐containing GSLs, are often used as ‘markers’ (e.g., GM1) of lipid rafts [9, 10] in cells of higher animals. Interestingly, gangliosides and sialic acid in general are not found in the GSLs of human parasites.

The *de novo* pathway of ceramide biosynthesis in animal cells involves the generation of the sphingoid base, a type of sphingosine, which is derived from palmitoyl CoA through the condensation with serine (Fig. 1) [11, 12, 13]. This generates dehydrosphingosine which is then converted to dihydrosphingosine (also called sphinganine). Acylation of dihydrosphingosine by fatty acid acyl CoA generates dihydroceramide which is desaturated to ceramide. The pathways of ceramide biosynthesis in parasites, while not broadly studied, appear similar in many ways, as shown for the protozoan parasite *Entamoeba histolytica*, which causes amebiasis in humans [14, 15], and the obligate intravacuolar protozoan parasite *Toxoplasma gondii*; interestingly, the latter can also salvage ceramide from its hosts [16]. In addition, many parasites have efficient salvage pathways for reutilizing sphingolipid precursors [13]. In animals, ceramide may also arise from degradation of sphingomyelin.

The ceramide may be glycosylated by enzymes that may add glucose, as for glucosylceramide, or galactose, as for galactosylceramide, and these may be further modified by additional sugars and by modifications with aglycone substituents, such as sulfation or phosphorylcholine (PC). The structure of the aliphatic chain of the sphingoid base, while often C_18_, can vary in alkyl chain length and/or branching, the numbers and positions of double bonds, and positions of hydroxyl groups, and the acyl chain can also vary greatly. These lipid moieties may affect trafficking of the ceramide and its accessibility to glycosyltransferases but that is not well understood. A single type of GSL with a single glycan structure may be expressed as a collection of glycolipid forms that vary in the lipid moiety. Often, unless the collection of intact GSLs is completely characterized by mass spectrometry and other methods, the emphasis is on the structure of the glycan moiety, and the structure of the ceramides may not be well characterized. This is especially true for many parasite‐derived GSLs.

**Fig. 2:**
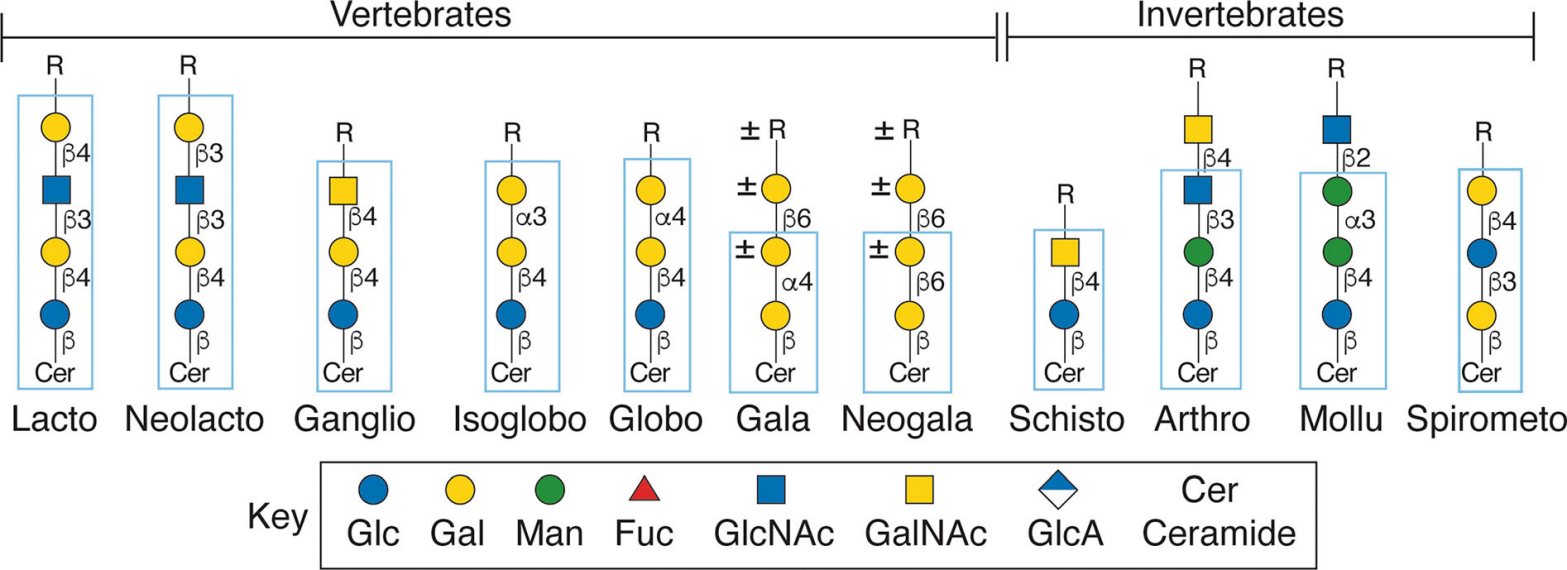
Examples of major core structures of GSLs found in vertebrates and invertebrates. The core structures bounded by the blue rectangles are defined as unique linkages of sugars to ceramide (Cer) and form the basis for a series of GSLs based on that core.

In regard to the glycan modification of ceramide, there are many types of core structures [17], but parasites can also synthesize unique types of core structures on their GSLs that are not found in vertebrates. Many of the defined core structures of GSLs, also termed series, are depicted in Fig. 2. Many of the GSL cores are discussed below for different parasites, but additional references for the GSL core sequences can be found here [17, 18], along with those for Neogala [19, 20], Mollu [21], and Arthro [22] not typically found in parasites.

## Analytical procedures for defining GSLs

There are many different approaches to define the structures of GSLs [23, 24, 25, 26]. GSLs are generally classified for analysis as either neutral or acidic. The latter types may contain uronic acids, sialic acids, sulfate, phosphate, PC, methylaminoethylphosphonate, etc. In general, due to their unusual properties, solvent‐based methods are used to extract GSLs from the organism, cells, or tissues, and either the intact GSLs or the free glycans released by enzymatic or chemical treatments are analyzed. Earlier methods of analysis involved thin‐layer chromatography or column chromatography to separate the glycolipids and identify differences in the lipid and/or glycan moieties. Modern analyses utilize hydrophilic interaction liquid chromatography coupled to electrospray ionization linear ion trap tandem mass spectrometry (HILIC‐ESI‐LIT‐MS/MS) [25]. Also, some studies use laser desorption/ionization time‐of‐flight mass spectrometry (MALDI‐TOF‐MS) (Fig. 1) as well as tandem mass spectrometry (MALDI‐TOF‐MS/MS) [27] with or without quantification and labeling with specific tags.

For GSLs, there are endohydrolases, termed endoglycoceramidases (or EGCs), such as endoglycoceramidase II, that can cleave the glycan from the ceramide. The released glycan has a reducing terminus and may be used for analysis by common glycomic‐based strategies. Most of these EGCs are active toward GSLs with the basic core of Glcβ1Cer (Fig. 2); it is uncertain whether all GSL core structures as found in many parasite GSLs are substrates for such endoglycosidases. Interestingly, EGCase III (now termed endogalactosylceramidase, EGALC) is specific for the 6‐gala‐series of GSLs [28] (Fig. 2). The EGCs were originally discovered in the 1980s from actinomycetes [29] and from leeches [30]. These enzymes have helped to revolutionize studies on GSL glycan structures, and many new enzymes have now been discovered [31]. General information about glycolipids and their analyses can be found at the Lipid Maps project https://www.lipidmaps.org/resources/tools/lm_software. Of note is that this site does not include specific information about GSLs in parasites, but focuses more on mammalian cells.

## GSLs in protozoan parasites

Protozoans are single‐celled organisms, and many of the protozoal parasites have complex glycomes, especially in regard to lipid‐related glycomolecules. Two such large classes in both protozoans and helminths are the glycolipids, such as GSLs, and the GPI‐anchored glycoproteins. Although not a focus of this review, it is interesting to note that the GPI‐anchored glycoproteins, which occur in protozoans and higher animals, were first well identified and their biosynthesis was characterized in trypanosomes [32]. They are a major form of cell‐surface glycoconjugates in protozoans in general, and in those organisms, they often dwarf the general glycome represented by other glycoconjugates. Such GPI anchors may be novel targets of immunity and vaccines in the future [6, 33]. Although the GSLs within protozoal parasites are not as well studied across all types of such parasites, they are generally very complex in structure and appear to be essential for pathogenesis.

## GSLs in *Plasmodium falciparum*

*Plasmodium falciparum*, which causes malaria, is an apicomplexan parasite, meaning it is a protozoan parasite with a variety of specialized organelles at the apical end which are used for invading cells. Another example of an apicomplexan parasite is *To. gondii*, which causes toxoplasmosis. While earlier studies had suggested that the late‐asexual stage of *P. falciparum* was unable to *de novo* synthesize GSLs, later studies demonstrated that *P. falciparum* can synthesize GSLs; the methods involved metabolic‐radiolabeling approaches using different stages of the parasite [34]. Other studies demonstrated that newly synthesized GSLs were detectable in the parasites incubated with [^3^H]serine and [^3^H]glucosamine [35]. Similar experiments were conducted on *To. gondii*, which also could incorporate these radioactive precursors into GSLs. Metabolic radiolabeling and the testing of different stages in parasite life cycle are often critical in such experiments, as it also can rule out contamination by nonparasite sources of the GSLs. The major GSL detected by such radiolabeling methods was monogalactosylcerebroside, but other glycolipids were also generated, including monogalactosyldiacylglycerol, and digalactosyldiacylglycerol (DGDG) [35].

Interestingly, the intraerythrocytic stages of the malarial parasite have enzymes to synthesize GSLs and can modify dihydroceramide *in vitro*. *De novo* synthesis of GSLs was documented by metabolic incorporation of ^14^C‐palmitic acid and ^14^C‐glucose in the three intraerythrocytic stages of the parasite, leading to production of monohexosylceramide, trihexosylceramide, and tetrahexosylceramide as analyzed by UV‐MALDI‐TOF mass spectrometry [34]. In addition, the inhibitor of GSL biosynthesis, d,l‐*threo*‐Phenyl‐2‐palmitoylamino‐3‐morpholino‐1‐propanol (PPMP), can inhibit GSL biosynthesis in parasite cultures, leading to a correlation between the arrest of parasite growth and inhibition of GSL biosynthesis, suggesting that the malarial glucosylceramide synthase may be a new target for malarial chemotherapy [36].

These metabolic studies have been strengthened by studies using monosaccharide derivatives. For example, the incorporation of N‐AzGlcNH_2_ in glycolipids of the intraerythrocytic stages of *P. falciparum* has been observed, and the MS analysis indicated the azido sugars were present within tetra‐ and pentasaccharides linked to ceramide [37].

While *P. falciparum* does not synthesize gangliosides, they can be acquired by the parasite during its complex binding, invasion and internalization, and production in infected erythrocytes. Recently, the glycosphingolipid GM3 has been suggested to be present, using quick‐freezing and freeze‐fracture immuno‐electron microscopy, in both the exoplasmic and cytoplasmic leaflets of *P. falciparum* malaria parasite plasma membrane [38]. This is may be the first example of a ganglioside facing the cytoplasmic side of the membrane.

## GSLs in *Toxoplasma gondii*

*Toxoplasma gondii* is an obligate intracellular protozoal parasite that causes toxoplasmosis, often acquired from uncooked meat; infections in people may last for years, and lead to serious health outcomes, especially in people with compromised immune systems. Some obligate protozoan parasites, such as *To. gondii*, may acquire or salvage their ceramide from the cells it infects in addition to synthesizing their own by *de novo* pathways [16, 39]. But most parasites appear to have the ability to generate ceramide *de novo*. While there is not much known about the GSLs in *To. gondii*, earlier studies indicated that the tachyzoites of this parasite could be identified by metabolic labeling of parasites with ^3^H‐serine and ^3^H‐galactose [39]. These glycolipids were characterized as sphingolipids based on the labeling protocols, and their insensitivity toward alkaline treatment. Synthesis of parasite GSLs was also inhibited by PPMP, and the GSLs identified were insensitive to treatment with endoglycoceramidase II; the authors suggested they might belong to globo‐type GSLs.

## GSLs in *Leishmania* spp.

The trypanosomatid parasites of the genus *Leishmania* are protozoans that cause visceral leishmaniasis (kala‐azar), which is transmitted through the bite of sandflies and can result in skin ulcers, disfiguring mucocutaneous lesions, and life‐threatening visceral infections [40]. A major type of glycolipid made in such parasites is lipophosphoglycans (LPGs), which can incite pro‐inflammatory and immunosuppressive innate immune responses in infected individuals [5]. These do not contain ceramide but are phosphatidylinositol‐anchored and have repeats of mannose phosphate, galactose, and glucosamine with additional modifications. Early studies suggested that *Leishmania amazonensis* can express GSLs in the amastigote forms, including those that are immunogenic and have the basic structure of the globo series, such as Galβ3Galα4Galβ4Glcβ1‐Cer [41] (Fig. 1). Also, monoclonal antibodies (mAbs) to this GSL could inhibit macrophage invasion by the parasite. Prior studies indicated that mAbs to *L. amazonensis* amastigotes recognized two GSLs with predicted compositions similar to the gangliosides GM1 and GD1a [42]. Biosynthetic studies at that time also indicated that labeled lipids could be incorporated into such GSLs localized on the surface of *L. amazonensis* amastigotes. Clearly, more studies are needed with modern technologies to explore the interesting GSLs of Leishmania.

## GSLs in parasitic helminths

The above concerned GSLs in single‐celled protozoan parasites. The other major class of parasites are multicellular ones termed helminths—they include trematodes (flatworms including platyhelminthes), nematodes, cestodes (tapeworms), and the intestinal horny‐headed worms (acanthocephalans), such as *Pomphorhynchus laevis*. Little is known about the GSLs in acanthocephalans, although they have a very complex glycocalyx [43].

## GSLs in *Schistosoma* spp.

*Schistosoma* spp. are parasitic blood trematodes, that infect people and many mammals [5]. The three major species that infect humans are *S. mansoni*, *S. japonicum*, and *S. haematobium*. They have a complex life cycle, which includes the adult stage as male/female worm pairs in people and animals, where eggs are produced daily. The eggs pass from the circulation into the digestive tract and then enter the infected host's feces. There they may gain access to fresh water. Each species of Schistosomes has a specific intermediate snail hosts. The infection of such permissive snails eventually results in the release of free‐swimming cercariae that may infect warm‐blooded animals venturing into the infected water, and the cycle continues, as the cercariae penetrate the skin of the vertebrate host and transform into schistosomula, a larval stage preceding maturation to adulthood.

Infected animals can develop immune responses to the glycans synthesized by the schistosomes, though depending on the vertebrate host, the strength of immunity varies tremendously [44, 45, 46, 47, 48, 49]. The parasites produce many unusual N‐ and O‐glycans in surface and secreted glycoproteins, and generate many types of GSLs. Many of the complex glycans of schistosomes have unusual linkages and sequences, including glycans with the LacdiNAc (GalNAcβ4GlcNAc‐R; LDN) motif, as well as highly fucosylated glycans, such as those containing the Lewis X antigen and repeating Lewis X, along with other unusual highly fucosylated glycans [5]. It is important to note that worms in general are lacking in sialic acid and their glycoconjugates are nonsialylated. The schistosome glycans are generated by a wide variety of enzymes encoded in the unique genome of these parasites; the generated glycans have many varied functions including immunological recognition and modulation of immunity [48, 50, 51, 52, 53, 54].

**Fig. 3:**
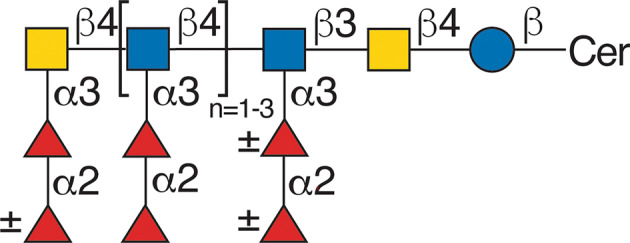
Example of a highly fucosylated GSL identified in the eggs of *Schistosoma mansoni*. See Khoo *et al*. [59] as cited in the text. See Fig. 2 for the key to the symbols.

*Schistosoma* spp. lack the ability to generate the lacto or neolacto series of GSLs, or any other core structures based on lactosylceramide, as the organisms lack the β1,4‐galactosyltransferase necessary for the core structure that acts typically on the precursor Glcβ1Cer; instead, they have a novel β1,4‐*N*‐acetylgalactosaminyl transferase that acts on glucosylceramide to generate the schisto core GalNAcβ4Glcβ1Cer (Fig. 2) [51, 55]. The parasites use this core to generate a wide variety of GSLs, containing the LacdiNAc sequence, chitin‐like sequences, and highly unusual poly‐fucosylated glycans [27, 56] (Fig. 3).

Several of the GSLs containing the schisto core of *S. mansoni* and other glycan types have been chemically synthesized in elegant studies by Kanaya *et al*. [57, 58]. The GSLs of the eggs of *S. mansoni* and *S. japonicum* contain large complex, primarily neutral GSLs, including large structures containing the Schisto core and the lacdiNAc antigen, along with novel fucosylation [59], which extend the earlier studies along this line [60].

The free‐swimming cercariae of *S. mansoni* contain relatively unique GSLs compared with the adult male/female worms. The cercariae also contain unusual complex GSLs built upon the schisto core and often containing the lacdiNAc sequence, and these include GalNAcβ4Glcβ1Cer; GlcNAcβ3GalNAcβ4Glcβ1Cer; Galβ4GlcNAcβ3GalNAcβ4β1Cer; Galβ4[Fucα3]GlcNAcβ3GalNAcβ4β1Cer (Lewis X pentasaccharide structure); Galβ4[Fucα3]GlcNAcβ3GlcNAcβ3GalNAcβ4β1Cer (Lewis X hexasaccharide structure); and Fucα3Galβ4[Fucα3]GlcNAcβ3GalNAcβ4β1Cer (pseudo‐Lewis Y hexasaccharide structure) [61].

## GSLs in *Brugia malayi*

Nematodes are roundworms, and many species are known to infect people and animals. While the glycoprotein glycans of nematodes can be unusual, the GSLs are also unlike those in mammals. They differ in core structures, extensions, and aglycone modifications such as addition of PC. As an aside, it should also be noted that some nematodes can also produce extraordinary glycolipids (ascarosides), such as ascarylose (didesoxymannose) sugar coupled to short (three‐ to six‐carbon) aliphatic side chains [62].

**Fig. 4:**
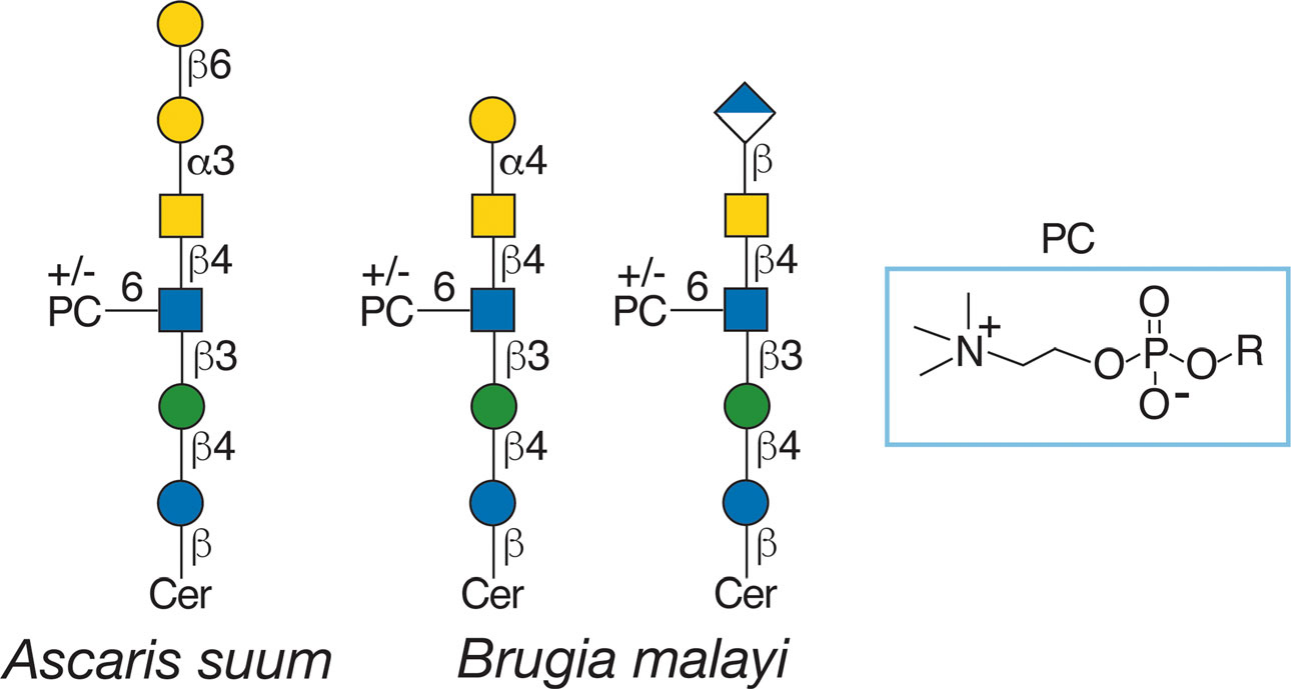
Examples of zwitterionic GSLs found in parasitic nematodes *Ascaris suum* and *Brugia malayi*. These examples exhibit different termini, either Gal or GlcA, and with or without PC modifications. See Fig. 2 for the key to the symbols.

Elegant studies on the glycome of the filarial nematode *B. malayi* identified many novel glycolipids [46]. MALDI‐TOF‐MS analyses revealed dozens of different GSLs, based on the arthro series GlcNAcβ3Manβ4Glc. Many novel terminal sequences have been identified including terminal α4‐linked galactose (Gal), PC modified GlcNAc residues, glucuronylated (GlcA) termini, and lacdiNAc motifs (Fig. 4) along with novel fucosylated (Fuc) termini. An especially important aspect of these studies is that the authors utilized many of the glycans from GSL, N‐, and O‐glycans to generate a glycan microarray, which allows easy interrogation of each glycan with antibodies in sera, such as that from *B. malayi*‐infected macaques. Studies using this approach led to the identification of both IgG and IgM antibodies to many GSL antigens above; remarkably, antibodies to the GSL antigens dominate over those to N‐glycan antigens and correlate with being induced by infections. By contrast, some antibodies, such as those to α‐linked galactose, are also present in uninfected macaques. There are also many similarities to the immune responses identified in infected macaques compared with *B. malayi*‐infected patients who were treated with the anthelminthic diethylcarbamazine citrate. Overall, the studies reveal that there are significant IgG and IgM induction to GSL glycans *B. malayi* upon infection, while binding to N‐glycans is comparatively weaker, particularly for IgM.

Recent studies using glycan microarray analyses also indicate that sera from individuals infected with *Onchocerca volvulus* or *Mansonella perstans* contain IgG (predominantly IgG1 and IgG2) antibodies to GSLs from *B. malayi* [63], and that these parasites share many structural features of their GSLs with those in *B. malayi*. Many of the arthro series GSLs from *B. malayi* contain the PC modification, but interestingly, this does not appear to greatly influence IgG binding to many of the glycans containing the terminal GlcA modification.

## GSLs in *Onchocerca volvulus*

*Onchocerca volvulus* is a filarial nematode and the cause of Onchocerciasis (river blindness); it is spread among people through the bites of female blackflies of the genus *Simulium*. Studies in this parasitic nematode were prompted in part by other studies demonstrating cross‐reactive GSLs between those in other parasitic nematodes that are not filarial, such as *Ascaris suum*, *Setaria digitata*, and *Litomosoides sigmodontis* [64]. The GSLs in *O. volvulus* are partly zwitterionic and contain PC modifications, which is a part of the cross‐reactive epitope. *A. suum* contains many unusual PC‐modified GSLs, such as Galα3GalNAcβ4[PC‐6]GlcNAcβ3Manβ4Glcβ1Cer (termed component A) and another Galα3GalNAcβ4[PC‐6]GlcNAcβ3[PE‐6]Manβ4Glcβ1Cer (termed component C), with PC and phosphoethanolamine (PE) substituents [65, 66]. In *O. volvulus*, two of the zwitterionic GSLs were identified as PC‐6GlcNAcβ3Manβ4Glcβ1Cer and GalNAcβ4[PC‐6]GlcNAcβ3Manβ4Glcβ1Cer and Galα3GalNAcβ4[PC‐6]GlcNAcβ3Manβ4Glcβ1Cer. Recent studies have also reported the chemical synthesis of the nonreducing ends of the GSLs in *A. suum* [67]. These include five different glycans, the largest of which contained the terminal sequence GlcNAcβ6Galβ6[Galβ3]Galα3GalNAcβ1‐R.

## GSLs in cestodes

Glycosphingolipids occur in cestodes (tapeworms), but only a few species have been studied in detail. Tapeworms are particularly pathogenic and cause common infections in people and their animals, and occur most commonly in areas of poor sanitation and where animals and people are concentrated.

## GSLs in *Spirometra erinaceieuropaei* and *Diphyllobothrium hottai*

*Spirometra erinaceieuropaei* is a parasitic tapeworm that mainly infects animals, and humans are rarely infected, but they may acquire the parasite from undercooked or contaminated meat. The GSLs from this cestode contain the unusual Spirometo core [68, 69] (Fig. 2). This core structure may be significant from a taxonomic sense, as perhaps characteristic of pseudophyllidean tapeworms. The GSLs of *S. erinaceieuropaei* contain novel structures, such as the unusual fucosyltetrahexosylceramide Galβ4(Fucα3)Glcβ3GalβCer identified in the plerocercoids (infective larvae) [68]. Ten GSLs were isolated from *D. hottai* adult worms and four from plerocercoids, comprising mono‐, di‐, tri‐, tetra‐, and pentasaccharides. The GSL Galβ4(Fucα3)Glcβ3Galβ1‐Cer was found in adult worms but not in plerocercoids, whereas Galβ4 (Fucα3)Glcβ3(Galβ6)Galβ1‐Cer was found in both adult worms and plerocercoids [70].

## GSLs in *Taenia* spp. and *Echinococcus* spp.

Eating uncooked meat can lead to infections and taeniasis in humans, which is caused by *Taenia saginata* (beef tapeworm), *T. solium* (pork tapeworm), and *Taenia asiatica* (Asian tapeworm). Tapeworms may infect the intestines and other organs. Another dangerous tapeworm is *E. multilocularis* acquired by swallowing the eggs of the parasite. Infection leads to causes alveolar echinococcosis. Cystic echinococcosis or hydatid disease is caused by infection with *Echinococcus granulosus*. The tapeworm can grow to ~ 2–7 mm long and can be found in dogs, which are the definitive host, and also found in sheep, cattle, goats, and pigs (intermediate hosts). While human infections may be asymptomatic, CE is associated with harmful, slowly enlarging cysts that arise in the liver, lungs, and other organs and may go unnoticed for years.

Early studies on the GSLs in the metacestodes of the fox tapeworm *Taenia crassiceps* found them to contain the neogala series, for example, Galβ1Cer, Galβ6Galβ1Cer, and Galβ6Galβ6Galβ1Cer [71]. Several of the neutral GSLs from metacestodes of *E. multilocularis* were identified in to be similar in the core sequence and contain Galβ1Galβ1Cer, Fucα3Galβ6Galβ1‐Cer, Galβ6Galβ6Galβ1‐Cer, and Galβ6[Fucα3]Galβ6Galβ1Cer [72] and have been synthesized [73]. Such glycans are highly immunogenic in infected patients with alveolar hydatid disease [74]. Biotinylated versions of these glycans have been synthesized recently and examined for recognition by antibodies in the sera of 60 patients with alveolar echinococcosis [75]. Among the biotinylated glycan tested, antibodies in the sera of alveolar echinococcosis patients differed in their recognition of the GSLs, raising the potential to use such glycans in diagnostics for this parasitemia.

## Concluding remarks

While much remains to be learned about the parasite GSLs, they exhibit remarkable diversity, both in core structures and in the types of extensions and glycan modifications, including aglycone moieties. Because by nature the GSLs are in parasites, the materials for such studies are often difficult to acquire, making broad‐based studies on the structure and biology of such GSLs difficult. This review has highlighted some of the more recent studies, and other historical studies in this field, in the hope that investigators will be invigorated to explore this important aspect of glycobiology. Understanding the structure, biosynthetic pathways, and functions of GSLs in parasites offers opportunities to develop new diagnostics and therapeutics, as many of the enzymes involved are novel and unrelated to host or mammalian GSLs. In addition, much remains to be learned about the functions of GSLs in parasites and their likely importance in host–parasite interactions.

## Conflict of interest

The author declares no conflict of interest.

## Author contributions

RDC wrote the article and prepared all figures.
